# Influence of Chromium Propionate Supplementation in Ewes During the Gestational Phase on the Regulation of Genes Associated with Growth and Metabolism in the Offspring

**DOI:** 10.1007/s12011-026-05185-4

**Published:** 2026-06-18

**Authors:** Daniela Lázara de Almeida, Helena Viel Alves Bezerra, Flávia Mallaco Moreira, Paulo Roberto Leme, Luis Orlindo Tedeschi, Heidge Fukumasu, Sarita Bonagurio Gallo

**Affiliations:** 1 Faculty of Veterinary Medicine and Animal Science, University of São Paulo, Pirassununga, São Paulo, 13635-900 Brazil; 2 Faculty of Animal Science and Food Engineering, University of São Paulo, Pirassununga, São Paulo, 13635-900 Brazil; 3 Department of Animal Science, Texas A&M University, College Station, TX 77843-2471 United States of America

**Keywords:** Fetal programming, Growth, Maternal nutrition, Trace mineral

## Abstract

Maternal chromium (Cr) supplementation has been associated with altered offspring growth, carcass traits, and tissue gene expression in several species, but underlying programming mechanisms in sheep remain unclear. This study evaluated the effects of late-gestation and early-lactation dietary chromium propionate in ewes on the expression of genes related to myogenesis and lipid metabolism in the progeny. Sixty-nine Dorper × Santa Inês ewes were assigned, in a randomized block design, to receive 0 (CR0), 0.5 (CR0.5), or 1.5 mg/day (CR1.5) of chromium propionate per ewe from the last 50 days of gestation through the 80 days of lactation. Lambs received no supplemental Cr. After weaning, 18 single-born male lambs (approximately 80 days of age; 31.64 ± 6.07 kg) were finished in a feedlot for 52 days and slaughtered at 47.39 ± 7.15 kg. Samples of longissimus muscle, liver, and subcutaneous adipose tissue were collected for RT-qPCR analysis of MYOD, FASN, LPL, LIPE, IGF1, and IGFBP2. Maternal Cr supplementation altered (*P* < 0.05) offspring gene expression in a tissue- and dose-dependent manner. The CR1.5 treatment downregulated MYOD, FASN, LIPE, and IGF1 in skeletal muscle and liver, while upregulating LPL in muscle and IGFBP2 in liver (*P* < 0.05). In adipose tissue, LPL was upregulated by CR1.5, and LIPE by CR0.5 (*P* < 0.05). These changes are consistent with enhanced lipolysis in adipose tissue and a redirection of energy toward muscle deposition. In conclusion, chromium propionate administered to ewes during late gestation and lactation promotes fetal programming by modulating the expression of myogenic and lipid-metabolism genes in the liver, skeletal muscle, and adipose tissue of the offspring.

## Introduction

Maternal nutrition during gestation plays a critical role in fetal development and can exert long-term effects on growth, metabolism, and productive performance of the offspring. This phenomenon, known as fetal programming, occurs when nutritional or environmental stimuli during critical windows of development induce persistent changes in tissue structure, physiology, and gene expression [1]. These effects are often mediated by epigenetic mechanisms, including DNA methylation, histone modifications, and chromatin remodeling, which regulate gene transcription without altering the underlying DNA sequence [2].

Among nutrients that can influence fetal programming, chromium (Cr) has received attention for its role in enhancing insulin sensitivity and glucose utilization. Cr potentiates insulin signaling by increasing insulin receptor activity and promoting downstream pathways involved in glucose uptake and nutrient metabolism [3, 4]. Through these effects, Cr may alter the intrauterine metabolic environment, thereby influencing the expression of genes associated with growth, energy metabolism, and tissue development in the offspring.

In addition to its metabolic role, Cr has been shown to modulate gene expression directly. Chromium supplementation has altered the expression of genes involved in lipid synthesis, glucose metabolism, and antioxidant defense, including acetyl-CoA carboxylase 1 (ACC1), peroxisome proliferator-activated receptors (PPARs), glucose transporter 4 (GLUT4), insulin-like growth factor 1 (IGF1), and genes related to insulin signaling [5, 6]. These responses suggest that Cr may influence transcriptional activity through epigenetic mechanisms and changes in cellular nutrient sensing.

In ruminants, maternal supplementation with chromium propionate, an organic Cr source with high bioavailability, has been associated with improvements in maternal metabolic status and positive effects on offspring performance, including changes in body weight, carcass characteristics, meat quality, bone development, and reproductive traits [7–10]. However, despite these productive responses, little is known about whether maternal chromium supplementation modifies the expression of genes involved in growth and metabolic regulation in the progeny.

We hypothesized that supplementation of ewes with chromium propionate during gestation and lactation would alter the expression of genes associated with growth and metabolism in their lambs as a consequence of fetal programming. Therefore, the objective of this study was to evaluate the effects of maternal chromium propionate supplementation on the expression of selected genes related to growth and metabolic pathways in lambs.

## Materials and Methods

### Ewes

This study was carried out at the experimental facility located on the Sheep Farming Sector of the Fernando Costa Campus, University of São Paulo (USP), Pirassununga, SP, Brazil. All experimental procedures were authorized by the Ethics Committee on Animal Experimentation (Approval No. 3126111220 (ID 009018)) and divided into two phases. Firstly, the initial phase covered only sheep matrices, and the second phase, only the progeny.

Sixty-nine crossbreed Dorper X Santa Inês pregnant ewes were used, with an average weight and age of 60 ± 4 kg and 3 ± 1 years, respectively. The ewes had their estrus cycle induced by male effect, during a continuous period of 52 days, and then were naturally mated with two contemporary Dorper breed sires. Pregnancy diagnosis was performed by ultrasound, 50 days post-breeding season.

From 100 days of gestation, the females were allocated in a randomized block design and distributed into four experimental treatments, including different levels of chromium propionate (KemTRACE Chromium^®^, KEMIN^®^) as follows: Control (CR0, no Cr added), CR0.5 (0.5 mg Cr propionate/animal/day) and CR1.5 (1.5 mg Cr propionate/animal/day). Cr supplementation was offered as a premix with corn bran in a separate trough at a rate of 100 g/animal/day to ensure complete intake of the daily Cr propionate dose before the complete diet was consumed.

The ewes were kept in paddocks with access to shade, water and mineral supplements *ad libitum*. The NRC [11] recommendations for pregnant ewes were followed in formulating the total diet, which consisted of corn silage, ground corn, soybean meal, calcitic limestone and mineral salt (Table 1), supplied twice a day at 8 am and 2 pm. The ewes continued to receive the respective experimental diets until 80 days of lactation, when the lambs were weaned.

**Table 1:** Feed and chemical composition of the nutrition used in the ewes’ diet, creep feeding and feedlot

Feed, % dry matter	Ewes’ nutrition	Creep feeding	Feedlot
Corn silage	57.0		
Hay			15.0
Corn	25.0	63.0	65.0
Soybean meal	15.0	32.0	17.0
Limestone	1.0	2.5	1.5
Mineral^1^	2.0	2.5	1.0
Calcium chloride^2^			0.5
Monensin sodium, mg/kg			4.0
Chemical composition, % dry matter			
Crude protein	12.55	19.50	15.33
Neutral detergent fiber	39.92	10.60	21.89
Acid detergent fiber	25.88	5.30	14.23
Mineral material	6.64	9.24	6.65
Ethereal extract	2.45	3.10	1.78
Calcium	1.07	1.84	1.51
Phosphorus	0.30	0.60	0.29

^1^ Guabiphos Ovinos AE® - Guabi Nutrição Animal (guaranteed levels per kg): calcium 140 g, phosphorus 65 g, magnesium 10 g, sulfur 12 g, sodium 130 g, cobalt 80 mg, iron 1000 mg, iodine 60 mg, manganese 3,000 mg, selenium 10 mg, zinc 5,000 mg, fluoride (max.) 650 mg, vitamin A 50,000 I.U., vitamin E 312 I.U.; ^2^ Nutricab – Kemin (assurance levels per kg): Calcium 275 g/kg, Chlorine 490 g/kg

### Offspring

From 20 days of age until weaning (80 days), lambs received concentrate ad libitum via creep feeding to ensure individual consumption without interference from the mothers. The concentrate formulation, common to all treatments, was composed of ground corn, soybean meal, limestone and mineral (Table 1). This standardization aimed to isolate the effects of maternal diet on the progeny, allowing the evaluation of fetal programming.

After weaning, 18 male, uncastrated lambs were transferred to the feedlot and allocated to one of the three treatments, totaling six replicates per treatment, where they were kept in individual pens (2 × 1.25 m) of a covered barn and fed twice daily, adjusted for *ad libitum* (5% leftovers) intake, as well as availability of water.

The lambs were fed with a diet composed of 15% roughage (*Cynodon* sp. hay) and 85% concentrate composed of ground corn, urea, soybean meal, limestone, minerals, calcium chloride and sodium monensin (Table 1). The diet was not altered during the confinement period to isolate the effects of maternal diet during pregnancy and lactation, enabling evaluation of fetal programming.

### Slaughter and Sampling

After 52 days of feeding, the lambs were carried to the slaughterhouse, located 200 m from the experimental barn, in a truck appropriate for sheep, after fasting from solids for a period of 16 h. The slaughter procedure followed the guidelines established by the Brazilian legislation [12]. Briefly, the stunning was performed with a penetrating captive bolt, followed by bleeding through the carotid artery and jugular vein. The carcasses were then skinned, eviscerated, washed, identified, and weighed.

### Gene Target Expression by Real-Time PCR

Immediately after removing the hide and evisceration, a sample of skeletal muscle was taken from the Longissimus dorsi muscle at the level of the 12th rib. This sample was promptly transported in liquid nitrogen and stored at -80 °C in an ultra-freezer for subsequent RNA extraction and quantitative Real-Time (RT-qPCR) analysis. Also, liver and visceral fat samples were collected using the same procedure.

The analyses were performed at the Laboratory of Comparative and Translational Oncology (LOCT) of the FZEA/USP. Total RNA was extracted using Trizol Reagent (Invitrogen Co., Carlsbad, CA, USA) according to the manufacturer’s instructions. RNA concentration was determined using a NanoDrop OneC spectrophotometer (Thermo Scientific, Carlsbad, CA, USA), and integrity was assessed by 28 S/18S rRNA band pattern on a 1% agarose gel. Isolated RNA was treated with DNase I (Thermo Scientific, Carlsbad, CA, USA) to remove genomic DNA.

cDNA synthesis was performed using the High-Capacity cDNA Reverse Transcription Kit (Applied BiosystemsTM, #4368814, Foster City, CA, USA) according to the manufacturer’s protocol. Primer pairs for target genes including myogenic differentiation 1 (MYOD1), fatty acid synthase (FASN), lipoprotein lipase (LPL), acetyl-CoA carboxylase alpha (ACACA), lipase E, hormone-sensitive type (LIPE), toll like receptor 2 (TLR2), insulin growth factor 1 (IGF1), insulin-like growth factor binding protein 2 (IGFBP2), and three reference genes such as actin beta (ACTB), heat shock protein 90 alpha class A and family member 1 (HSP90), and glyceraldehyde-3-phosphate dehydrogenase (GAPDH) were designed using the online software program Primer3Plus [13]. The specificity of the designed primers was evaluated using the PrimerBlast software from the NCBI database [14].

Quantitative mRNA analyses were performed on a CFX96TM thermocycler (Bio-Rad) in 10 µL reactions containing 2x qPCRBIO SyGreen Mix (PCRBiosystems, London, UK), 1 µl cDNA, and forward and reverse primers at optimized concentrations determined for each gene (Table 2). The cycling parameters for the FASN, IGF1, and IGFBP2 genes were 2 min at 95 °C followed by 40 repetitions of 5s at 95 °C and 30s at 60 °C. For the MYOD and LPL genes, the cycling conditions were 2 min at 95 °C followed by 40 repetitions of 5s at 95 °C, 30s at 60 °C, and 10s at 80 °C. LIPE gene expression was performed under the following conditions: 2 min at 95 °C followed by 40 repetitions of 5s at 95 °C, 30s at 60 °C, and 10s at 77 °C. Finally, the cycling conditions for the ACACA and TLR2 genes were 2 min at 95 °C followed by 40 repetitions of 5s at 95 °C, 30s at 60 °C, and 10s at 79 °C. All reactions were followed by melting curve analysis to confirm amplification of single cDNA products.

All samples were amplified in duplicate from the same RNA preparation, and the mean value was considered. RT-PCR efficiency was evaluated for each gene based on the slope of a linear regression model using a set of cDNA samples as PCR templates across a 3-fold dilution series. PCR amplification efficiency was calculated according to the equation E = 10(-1/slope) [14].

Relative quantification (RQ) values were determined by a variation of the Pfaffl E′ target ΔCt (calibrator Ct – Sample Ct) method [14]. Relative gene expression was normalized to the geometric mean of the transcription level of HSP90 and GAPDH (housekeeping genes) for liver and adipose tissues to identify differences between groups, as previously described [15]. In muscle tissue, HSP90 and ACTB were used as housekeeping genes (Table 2).

**Table 2:** Nucleotide sequence of PCR primers used to assay quantitative gene expression by real-time qPCR

Genes		Primers sequency (5’ – 3’)	Amplicon size (bp)	Final concentration in qPCR reaction
MYOD1	F	TGCTACGACCGCGCTTACTA	96	400 nM
	R	CACGATGCTGGACAGGCAGT		
FASN	F	AAGTCGAACATGGGACACCC	115	300 nM
	R	GGTTTGGGTTGTGGAAGTGC		
LPL	F	TGGAGTGACGGAATCTGTGG	150	300 nM
	R	ACGTTGGAGTCTGGTTCCCT		
LIPE	F	TCTCTGAAGAGGCCTGGGAG	101	600 nM
	R	AAGGCCATGTTGTCCTCTGC		
IGF1	F	TGTACTGTGCGCCTCTCAAG	126	300 nM
	R	TGTTTCCTGCACTCCCTCTG		
IGFBP2	F	GGTGGCAAACATCACCTTGG	150	400 nM
	R	ATAGAGGTGCTCCAGGGGAC		
ACTB	F	TCGTGGTTGACAACGGCTC	136	300 nM
	R	TAGGAGTCCTTCTGGCCCAT		
HSP90	F	AAGAGCCTGACCAACGACTG	104	300 nM
	R	GGAGCTCGTCTTGGGACAAA		
GAPDH	F	GGTGATGCTGGTGCTGAGTA	139	300 nM
	R	CCATCACAAACATGGGAGCG		

### Statistical Analysis

The data were analyzed in a randomized block design (ewe reproductive category: primiparous and multipara), with 18 animals (single-birth lambs) allocated to three treatments, yielding 6 replicates per treatment. Each lamb was considered an experimental unit. For the analysis of the effect of the treatments on the variables studied, the treatments were treated as fixed effects and the blocks as random effects. The means were compared using Tukey’s test (*p* < 0.05).

The mathematical model was described:

$${\mathrm Y}_-\mathrm{ij}\;=\;\mathrm\mu\;+\;{\mathrm t}_-\mathrm i\;+\;{\mathrm b}_-\mathrm j\;+\;{\mathrm e}_-\mathrm{ij}$$

Where:

Y_ij = value of the parcel that received treatment i in block j;

µ = overall mean;

t_i = effect of treatment i, where i = 3 ;

b_j = effect of block j, where j = 2;

e_ij = error associated with the parcel that received the treatment i in the block j.

The data were analyzed using analysis of variance with the MIXED procedure in SAS 9.4 [16].

## Results

**Fig. 1:**
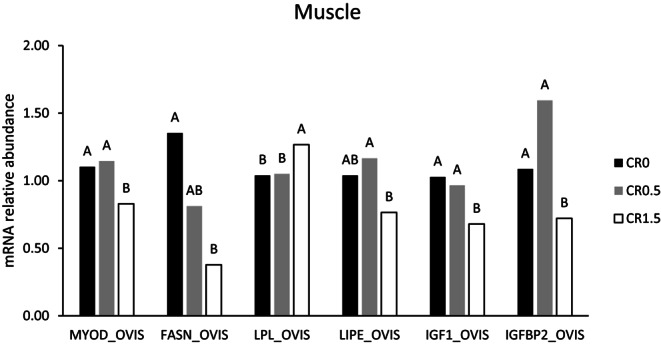
Relative expression levels of target genes in skeletal muscle of lambs from ewes fed different concentrations of chromium propionate. Legend: CR 0 - no addition of Cr propionate; CR0.5 - 0.5 mg of Cr propionate per animal/day; CR1.5 - 1.5 mg of Cr propionate per animal/day; MYOD – myogenic differentiation; FASN – fatty acid synthesizer; LPL – lipoprotein lipase; LIPE - lipase E, hormone-sensitive type; IGF1 - insulin growth factor 1; IGFBP2 - insulin-like growth factor. A-B Means in the same row with different superscript letters differ by Tukey's test (*P* < 0.05)

In the gene expression profile in skeletal muscle, detected by real-time PCR, the CR1.5 group showed lower expression for the genes MYOD, FASN, LIPE, IGF1 and IGFBP2. On the other hand, LPL expression was higher in the CR1.5 group (Fig. 1, *P* < 0.05).

**Fig. 2:**
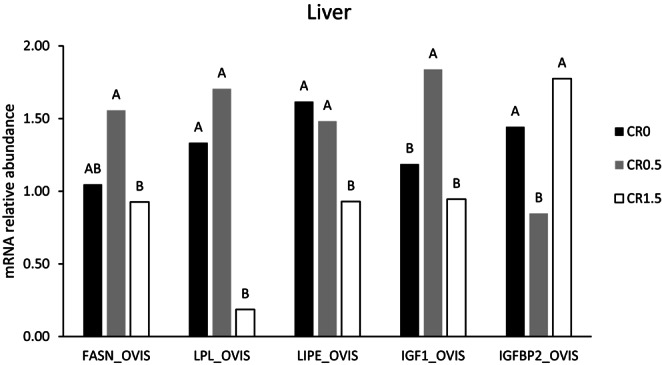
Relative expression levels of target genes in the liver of lambs originated from ewes fed different concentrations of chromium propionate. Legend: CR 0 - no addition of Cr propionate; CR0.5 - 0.5 mg of Cr propionate per animal/day; CR1.5 - 1.5 mg of Cr propionate per animal/day; MYOD – myogenic differentiation; FASN – fatty acid synthesizer; LPL – lipoprotein lipase; LIPE - lipase E, hormone-sensitive type; IGF1 - insulin growth factor 1; IGFBP2 - insulin-like growth factor. A-B Means in the same row with different superscript letters differ by Tukey's test (*P* < 0.05)

In the liver, higher FASN gene expression was observed in response to CR0.5 supplementation (*P* < 0.05). The CR1.5 treatment reduced the expression of the FAS, LPL, LIPE, and IGF1 genes in the liver (*P* < 0.05, Fig. 2). The influence of CR0.5 supplementation was observed in higher IGF1 expression levels. Regarding IGFBP2, lower expression levels were observed in the CR0.5 group (Fig. 2; *P* < 0.05).

**Fig. 3:**
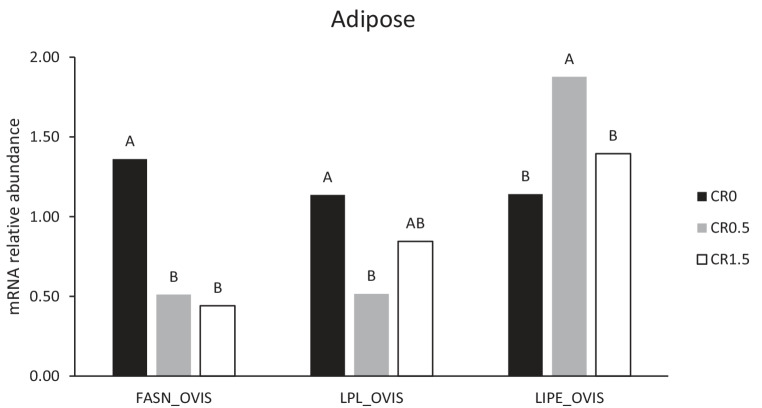
Relative expression levels of target genes in adipose tissue of lambs originating from ewes fed different concentrations of chromium propionate. Legend: CR 0 - no addition of Cr propionate; CR0.5–0.5 mg of Cr propionate per animal/day; CR1.5–1.5 mg of Cr propionate per animal/day; FASN - fatty acid synthesizer; LPL - lipoprotein lipase; LIPE - lipase E, hormone-sensitive type. A- B Means in the same row with different superscript letters differ by Tukey’s test (*P* < 0.05)

Regarding adipose tissue, the gene expression profile showed effects on the relative expression levels of FASN and LPL in the CR0 treatment and LIPE in the CR0.5 treatment (Fig. 3, *P* < 0.05). Cr treatments reduced the expression of FASN and LPL genes in adipose tissue (Fig. 3).

## Discussion

Increased muscling and deposition of lean tissue have been a consistent response to Cr feeding in farm animals, such as poultry [17, 18] and pigs [19, 20], as well as in some studies with lambs [21–23]. MYOD gene is known for its response to muscle differentiation, growth and development [24, 25]. However, in this study, greater expression was observed in the control group (without Cr propionate supplementation) and in the CR0.5 treatment, suggesting that it is not positively regulated or that it has an effect on muscle development at higher dosages in lambs originating from fetal programming. This may suggest that Cr supplementation doses in the ewe’s diet, focusing on fetal programming, should be less than 1.5 mg/kg of dry matter.

It may seem paradoxical; however, MYOD acts during myogenic differentiation in the fetal phase, converting precursor cells into myoblasts together with Myf-5, a myogenic regulatory factor (MRF) [26]. Myogenin, another MRF, is required for myoblast fusion into myotubes and is expressed later and maintained throughout the fetal stage. MYOD and Myf-5 function in a compensatory manner to induce the differentiation of multipotent myogenic precursor cells into myoblasts [27]. These findings may suggest that during the fetal phase, a higher Cr dose may have directed energy toward myogenesis, leading to greater cell differentiation. This process is not necessary after the fetal phase. Lower dosages or non-supplementation are associated with greater regulation of MYOD expression in skeletal muscle, suggesting a compensatory increase in cell differentiation after the fetal phase, especially during the termination phase, when energy requirements are higher.

Similarly, greater FASN expression was observed in the skeletal muscle and liver tissues of the control and CR0.5 groups, and in the adipose tissue of the CR0.5 and CR1.5 groups. FASN (Fatty Acid Synthase) is known for its role in catalyzing the synthesis of fatty acids from sugars in the process of cellular respiration. Studies have linked FASN expression or activity to metabolic alterations in humans, such as obesity, dyslipidemia, insulin resistance, and an altered adipocytokine serum profile [28]. The expression of this enzyme is highly dependent on nutritional conditions in lipogenic tissues. FASN-catalyzed endogenous fatty acid biosynthesis in liver and adipose tissue is stimulated by a high carbohydrate diet [29]. In the present study, the lambs were fed a high-starch diet composed of 85% concentrate, which readily stimulated FASN activity/expression due to the high availability of circulating glucose, suggesting an inhibitory effect of Cr on FASN expression in the evaluated tissues.

The influence of higher Cr doses was also observed in the downregulation of LIPE, IGF1, and IGFBP2 in skeletal muscle and liver. Hormone-sensitive lipase (LIPE) catalyzes the release of fatty acids from triglycerides in adipocytes and cholesterol from cholesterol esters in steroidogenic tissues. That is, in the present study, a reduced lipolytic activity is observed in the skeletal muscle and liver tissues of lambs in the finishing phase, in response to Cr supplementation in the fetal phase. Lipolytic activity is generally controlled by dietary energy intake [30]. It suggests that energy production from glucose may reduce the activity of fatty acid β-oxidation processes, as found by [31], who supplemented lambs with 3 mg Cr/kg DM during the finishing phase, and similarly, observed a downregulation of genes related to lipid β-oxidation in the skeletal muscle tissue, as an epigenetic response to Cr supplementation.

On the other hand, LIPE was upregulated in the adipose tissue in the CR0.5 group. The sequential hydrolysis of triacylglycerol stored in fat cells defines the process of lipolysis. These target genes are regulated by the Peroxisome Proliferator Activated Receptor (PPAR) family, which executes multiple functions, especially in energy balance, cell differentiation, and lipid metabolism [32]. In ruminants, the PPAR isoform is the most abundant in skeletal muscle [33], whose activation can trigger lipid β-oxidation [34]. Indeed, in the present study, target genes associated with lipolytic activity were downregulated in skeletal muscle and liver tissues. Target genes associated with lipolytic activity were downregulated in skeletal muscle and liver tissues, suggesting that energy consumption, directed as glucose to the muscle and liver, makes lipid beta-oxidation unnecessary.

However, in adipose tissue, beta-oxidation became necessary in response to Cr supplementation during the fetal stage, as evidenced by higher expression of LPL and LIPE. In humans, adipocyte size is associated with the proportion of visceral fat and is increased in insulin-resistant subjects [35]. Curiously, in this study, a higher kidney fat weight was observed with CR1.5 supplementation [9]. Similarly, as reported by Bezerra et al. [31], who used a glycogenic diet (94% concentrate), excess glucose not consumed by muscle tissue may have been redirected toward the synthesis of fatty acids in visceral fat deposits.

Despite one of the main effects of Cr being the reduction of carcass fat due to changes in adipose tissue’s response to insulin, not all fat-depositing sites respond equally to this hormone [36]. A viable hypothesis relates to the antilipolytic effect of insulin on adipose tissue [37]. However, paradoxically, it seems that Cr supplementation in the fetal phase, despite redirecting the remaining glucose not used in other tissues to adipocytes in visceral fat, also acts as a compensatory mechanism in the termination phase at later life, increasing lipolytic activity in adipose tissue.

Furthermore, lower IGFBP2 expression was observed in response to Cr supplementation during the fetal phase, in both skeletal muscle and hepatic tissue. IGFBP2 is known for its role in encoding a protein, which is one of six similar proteins that bind insulin-like growth factors I and II (IGF-I and IGF-II). The IGF1R-targeted agents are safer than anticipated, with limited metabolic toxicity, mainly hyperglycemia. Cr is known for its effect on increasing glucose uptake in glucose/insulin-sensitive tissues. The regulation of glucose in skeletal muscle is a crucial aspect of ruminant physiology, where maintaining blood levels is linked to the deposition of proteins and fats in the body [38].

At the molecular level, studies such as that by Zhang et al. [39] in pigs reported that Cr supplementation acts in modulating RNA synthesis and regulating protein translation in skeletal muscle tissue. Also, Bezerra et al. [31] found upregulation of the RPL35A, RPL39, and RPS25 genes in the skeletal muscle of lambs supplemented with 3.0 mg Cr/kg DM, with a significant impact on ribosome and ribosome biogenesis pathways, as determined by KEGG functional analysis. Interestingly, the positive regulation of the RPL35A gene, which encodes ribosomal proteins, has been associated with hypertrophy of muscle mass in humans [26]. Indeed, in the present study, higher muscle deposition was observed in the leg, sirloin, and neck commercial cuts of lambs in response to Cr supplementation during the fetal phase [40].

Also, the liver is the major glycogen storage organ and plays a significant role in carbohydrate metabolism. Lopez et al. [40] evaluated the role of IGF-II in hepatic glycogen metabolism in normal and growth-retarded IGF-II-deficient mice. They found that IGF-II is required for the regulation of glycogen metabolism in the mouse during the perinatal period, possibly by stimulating glycogen synthase activity, as IGF-II-deficient mice showed significantly lower liver glycogen content on embryonic day 18 and postnatal day 0. The authors concluded that IGF-II may regulate fetal growth by perinatally regulating glycogen synthesis and plays an important role in the transition from fetal to postnatal life, as it protects the neonate against hypoglycemia.

Interestingly, in the present study [9], it was found that lambs whose mothers received CR1.5 had higher birth weight, an effect observed up to 45 days of age, in response to fetal programming. Apparently, Cr acted as an energy redirector to muscle tissue, where, instead of directing glucose to storage in the form of glycogen, it was directed to protein deposition.

## Conclusion

Maternal supplementation with chromium propionate, particularly at 1.5 mg/kg DM, promotes fetal programming in male sheep offspring by modulating genes associated with lipid metabolism and the IGF growth axis. In liver and adipose tissue, the CR1.5 treatment downregulated genes related to lipid synthesis and uptake, suggesting a potential reduction in adipogenic activity in the offspring. However, the decreased expression of myogenic genes and muscle IGF1 indicates that excessive maternal Cr supplementation may also alter pathways involved in muscle development. Therefore, although chromium supplementation appears effective in modulating metabolic pathways associated with reduced fat deposition, its dosage should be carefully optimized to avoid potential negative effects on muscle growth and development in the offspring.

## Data Availability

All the project data is in the article. Further information can be requested from the corresponding author.
