# Correction to: Influence of Chromium Propionate Supplementation in Ewes During the Gestational Phase on the Regulation of Genes Associated with Growth and Metabolism in the Offspring

**DOI:** 10.1007/s12011-026-05243-x

**Published:** 2026-07-21

**Authors:** Daniela Lázara de Almeida, Helena Viel Alves Bezerra, Flávia Mallaco Moreira, Paulo Roberto Leme, Luis Orlindo Tedeschi, Heidge Fukumasu, Sarita Bonagurio Gallo

**Affiliations:** 1 Faculty of Veterinary Medicine and Animal Science, University of São Paulo, Pirassununga, São Paulo 13635-900 Brazil; 2 Faculty of Animal Science and Food Engineering, University of São Paulo, Pirassununga, São Paulo 13635-900 Brazil; 3 Department of Animal Science, Texas A&M University, College Station, TX 77843-2471 USA

**Correction to** **:** **Biological Trace Element Research**

10.1007/s12011-026-05185-4

The published version of this article unfortunately contained a mistake.

In the original article, Figs. 1 and 2 are distorted. Below are the correct Fig. 1 and 2.

**Fig. 1:**
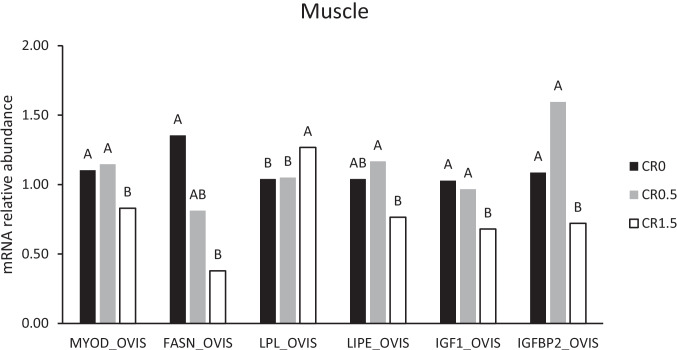
Relative expression levels of target genes in skeletal muscle of lambs from ewes fed different concentrations of chromium propionate. Legend: CR 0—no addition of Cr propionate; CR0.5—0.5 mg of Cr propionate per animal/day; CR1.5—1.5 mg of Cr propionate per animal/day; MYOD – myogenic differentiation; FASN – fatty acid synthesizer; LPL – lipoprotein lipase; LIPE—lipase E, hormone-sensitive type; IGF1—insulin growth factor 1; IGFBP2—insulin-like growth factor. A-B Means in the same row with different superscript letters differ by Tukey's test (P < 0.05)

**Fig. 2:**
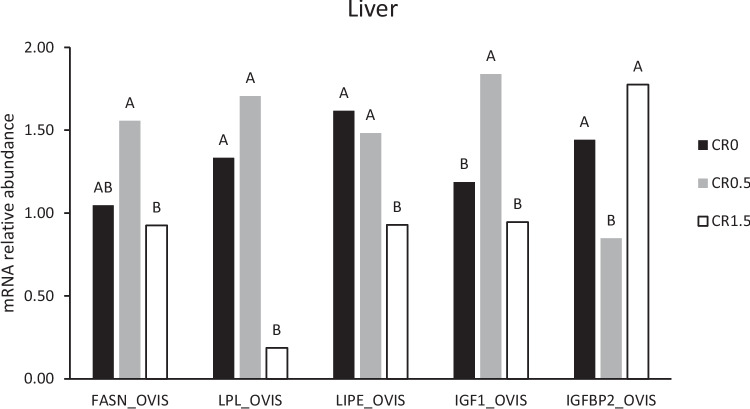
Relative expression levels of target genes in the liver of lambs originated from ewes fed different concentrations of chromium propionate. Legend: CR 0—no addition of Cr propionate; CR0.5—0.5 mg of Cr propionate per animal/day; CR1.5—1.5 mg of Cr propionate per animal/day; MYOD – myogenic differentiation; FASN – fatty acid synthesizer; LPL – lipoprotein lipase; LIPE—lipase E, hormone-sensitive type; IGF1—insulin growth factor 1; IGFBP2—insulin-like growth factor. A-B Means in the same row with different superscript letters differ by Tukey's test (P < 0.05)

The original article has been corrected.

The online version of the original article can be found at 10.1007/s12011-026-05185-4.

